# Dipeptidyl Peptidase IV Inhibition Exerts Renoprotective Effects in Rats with Established Heart Failure

**DOI:** 10.3389/fphys.2016.00293

**Published:** 2016-07-12

**Authors:** Daniel F. Arruda-Junior, Flavia L. Martins, Rafael Dariolli, Leonardo Jensen, Ednei L. Antonio, Leonardo dos Santos, Paulo J. F. Tucci, Adriana C. C. Girardi

**Affiliations:** ^1^Heart Institute (InCor), University of São Paulo Medical SchoolSão Paulo, Brazil; ^2^Cardiology Division, Department of Medicine, Federal University of São PauloSão Paulo, Brazil; ^3^Department of Physiological Sciences, Federal University of Espírito SantoVitória, Brazil

**Keywords:** vildagliptin, cardiorenal dysfunction, fluid retention, glucagon-like peptide-1, proteinuria, megalin

## Abstract

Circulating dipeptidyl peptidase IV (DPPIV) activity is associated with worse cardiovascular outcomes in humans and experimental heart failure (HF) models, suggesting that DPPIV may play a role in the pathophysiology of this syndrome. Renal dysfunction is one of the key features of HF, but it remains to be determined whether DPPIV inhibitors are capable of improving cardiorenal function after the onset of HF. Therefore, the present study aimed to test the hypothesis that DPPIV inhibition by vildagliptin improves renal water and salt handling and exerts anti-proteinuric effects in rats with established HF. To this end, male Wistar rats were subjected to left ventricle (LV) radiofrequency ablation or sham operation. Six weeks after surgery, radiofrequency-ablated rats who developed HF were randomly divided into two groups and treated for 4 weeks with vildagliptin (120 mg/kg/day) or vehicle by oral gavage. Echocardiography was performed before (pretreatment) and at the end of treatment (post-treatment) to evaluate cardiac function. The fractional area change (FAC) increased (34 ± 5 vs. 45 ± 3%, *p* < 0.05), and the isovolumic relaxation time decreased (33 ± 2 vs. 27 ± 1 ms; *p* < 0.05) in HF rats treated with vildagliptin (post-treatment vs. pretreatment). On the other hand, cardiac dysfunction deteriorated further in vehicle-treated HF rats. Renal function was impaired in vehicle-treated HF rats as evidenced by fluid retention, low glomerular filtration rate (GFR) and high levels of urinary protein excretion. Vildagliptin treatment restored urinary flow, GFR, urinary sodium and urinary protein excretion to sham levels. Restoration of renal function in HF rats by DPPIV inhibition was associated with increased active glucagon-like peptide-1 (GLP-1) serum concentration, reduced DPPIV activity and increased activity of protein kinase A in the renal cortex. Furthermore, the anti-proteinuric effect of vildagliptin treatment in rats with established HF was associated with upregulation of the apical proximal tubule endocytic receptor megalin and of the podocyte main slit diaphragm proteins nephrin and podocin. Collectively, these findings demonstrate that DPPIV inhibition exerts renoprotective effects and ameliorates cardiorenal function in rats with established HF. Long-term studies with DPPIV inhibitors are needed to ascertain whether these effects ultimately translate into improved clinical outcomes.

## Introduction

Dipeptidyl peptidase IV (DPPIV) is a widely expressed ectopeptidase that exists anchored as a transmembrane protein or in a soluble form in plasma and other body fluids (Lambeir et al., 2003). The kidney is the main source of DPPIV, where the enzyme is highly concentrated in the apical microvilli of the proximal tubule cells (Kenny et al., 1976; Girardi et al., 2001). DPPIV catalyzes the release of N-terminal dipeptides from polypeptides with proline or alanine in the second position. Among the many bioactive peptides cleaved by DPPIV, there is the gut hormone glucagon-like peptide-1 (GLP-1), which is the major incretin responsible for post-prandial insulin secretion. Indeed, DPPIV inhibitors, commonly called gliptins, increase the bioavailability of GLP-1 and improve systemic glucose homeostasis, thereby constituting the second line oral therapy in type 2 diabetes.

In addition to their effects on glycemic control, DPPIV inhibitors have been shown to produce cardioprotective and renoprotective effects. These beneficial actions may also be attributed, at least partially, to increased GLP-1 bioavailability. Upon binding of GLP-1 to its receptor (GLP-1R) in the heart, it induces activation of the cytoprotective signaling pathways cAMP/PKA and PI3K/Akt (Ussher and Drucker, 2012), reduces apoptosis (Ravassa et al., 2011), increases heart glucose uptake (Bao et al., 2011), reduces infarct size (Timmers et al., 2009) and improves coronary blood flow by means of its vasodilating actions (Ban et al., 2008). Moreover, GLP-1 induces diuresis and natriuresis by increasing renal blood flow and glomerular filtration rate (GFR) and by reducing NHE3-mediated sodium reabsorption in the renal proximal tubule sodium via cAMP/PKA activation (Crajoinas et al., 2011; Rieg et al., 2012; Farah et al., 2016).

Recent translational studies have suggested that increased DPPIV activity may play an important role in the pathophysiology of heart failure (HF) (Shigeta et al., 2012; dos Santos et al., 2013; de Almeida Salles et al., 2016). We have previously showed that increased circulating DPPIV activity is associated with worse cardiovascular outcomes in human and experimental HF and that long-term DPPIV inhibition exerts cardioprotective actions that prevent the development/progression of HF in rats (dos Santos et al., 2013; de Almeida Salles et al., 2016).

Worsening renal function in the setting of HF is widely accepted as an independent risk factor for a poor prognosis. HF is often associated with sodium and water retention, a reduction in renal blood flow and GFR (Laskar and Dries, 2003), renal tubular damage and proteinuria (Udani and Koyner, 2010; Boerrigter et al., 2013). Despite its natriuretic actions, DPPIV inhibitors have also been shown to confer renoprotection by reducing urinary protein excretion and ameliorating renal damage in a variety of clinical and experimental studies in diabetic, obese and renal failure patients and rodents (Hattori, 2011; Kanasaki et al., 2014; Scirica et al., 2014; Sharkovska et al., 2014; Nakamura et al., 2016; Tsuprykov et al., 2016). However, it remains to be established whether DPPIV inhibitors are capable of reversing cardiorenal dysfunction after the onset of HF.

Given the inextricable importance of heart-kidney interactions in HF, the main purpose of the present study was to test the hypothesis that DPPIV inhibition by vildagliptin improves renal water and salt handling and exerts anti-proteinuric effects in rats with established HF. The molecular mechanisms underlying these renoprotective effects were also investigated.

## Materials and methods

### Reagents and antibodies

The dipeptidyl peptidase IV (DPPIV) inhibitor vildagliptin was a gift from Novartis Pharmaceuticals (Basel, Switzerland). The monoclonal antibody (mAb) against the Na^+^/H^+^ exchanger isoform 3 (NHE3), clone 3H3 (Kocinsky et al., 2005), and a polyclonal antibody against megalin were kindly provided by Dr. Peter Aronson and Dr. Daniel Biemesderfer, respectively (Yale University, New Haven, CT). A mAb antibody against DPPIV (clone 5H8), a mouse mAb, clone 14D5, that recognizes NHE3 only when it is phosphorylated at serine 552 (Kocinsky et al., 2005) and polyclonal antibodies against the glucagon-like peptide-1 receptor (GLP-1R), cubilin, nephrin, and podocin were purchased from Santa Cruz Biotechnology (Santa Cruz, CA). The polyclonal antibody against phospho-(Ser/Thr) protein kinase A (PKA) substrates (Gronborg et al., 2002) was obtained from Cell Signaling (Beverly, MA). The mAb against actin (JLA20) was purchased from Merck Millipore (Darmstadt, Germany). A polyclonal antibody against CD31 was purchased from Abcam (Cambridge, MA). Horseradish peroxidase-conjugated goat anti-mouse, goat anti-rabbit and rabbit anti-goat secondary antibodies were purchased from Jackson ImmunoResearch (West Grove, PA). Chemicals were obtained from Sigma-Aldrich (St. Louis, MO) unless otherwise specified.

### Animal model

Experiments were carried out using male Wistar rats (2–3 months old, 200–250 g) obtained from the University of São Paulo Medical School, São Paulo, SP, Brazil. Animals were maintained in the Heart Institute animal facility in compliance with the Ethical Principles of the Brazilian College of Animal Experimentation and were approved by the Institutional Animal Care and Use Committee.

Experimental HF was induced by left ventricular (LV) myocardial injury after radiofrequency catheter ablation as previously described (Antonio et al., 2009; Inoue et al., 2012; dos Santos et al., 2013; de Almeida Salles et al., 2016). Briefly, rats were anesthetized with isoflurane, intubated and mechanically ventilated with oxygen enriched air and kept warm during surgical procedures (37⋅C). Subsequently, a left-side thoracotomy was performed at the fourth intercostal space. LV injury was created by delivering high-frequency currents (1000 KHz, 12 w, during 10 s) generated by a conventional radiofrequency generator (model TEB RF10; Tecnologia Eletrônica Brasileira, São Paulo, Brazil). The sham group underwent similar surgical procedures, but was not subjected to radiofrequency myocardial ablation. Six weeks after surgery, radiofrequency-ablated rats that developed HF were randomly divided into two groups and treated during 4 weeks with vildagliptin (120 mg/kg/day bid) or vehicle by oral gavage. Vehicle-treated sham rats were used as controls. Echocardiographic evaluation of left ventricle systolic and diastolic function as well as serum levels of BNP were used to characterize HF. HF was considered when the fractional area change (FAC) was lower than 40% and circulating levels of brain natriuretic peptide (BNP) were higher than 1.0 ng/mL. For determination of BNP serum concentration, blood samples were withdrawn from the rat retro-orbital sinus under isoflurane anesthesia and immediately transferred into chilled tubes containing clot activator and gel for serum separation (BD vacutainer® SST® II Advance®). Serum levels of BNP were measured by enzyme-linked immunoassay (ELISA) (BNP 32 Rat ELISA kit, Abcam) according to the manufacturer's instructions. Mortality rate between HF rats treated with vehicle or with vildagliptin was lower than 5% and did not differ between these two groups of rats.

### Echocardiography

Doppler echocardiography was performed 6 weeks after LV-radiofrequency ablation or sham surgery (pretreatment) and after treatment with vehicle or vildagliptin (post-treatment). Animals were anesthetized with ketamine and xylazine (50 mg/kg and 10 mg/kg, respectively, *i.p*.) and placed in the left lateral decubitus position (45⋅ angle) to obtain cardiac images. Images were captured and analyzed using Sonos 5500 ultrasound equipment (Philips Medical System, Bothell, WA) with a 12–14 MHz transducer (2 cm depth with fundamental and harmonic imaging). Echocardiographic images were acquired placing the cursor of pulsed wave Doppler in the LV outflow tract to display the end of aortic ejection and the onset of mitral inflow. End-diastolic (DA) and end-systolic (SA) transverse areas of LV were measured in parasternal-papillary short-axis images. FAC was calculated based on the areas using the following equation: FAC (in %) = (DA − SA)/DA × 100. Diastolic function was obtained at apical plane images. Isovolumetric relaxation time (IVRT) was measured in tissue Doppler images in the lateral wall of the ventricle. Transthoracic echocardiography was performed by two investigators who were blind to the experimental groups.

### Renal function

Renal function was evaluated as described previously (Inoue et al., 2013). In brief, rats were housed individually in metabolic cages (Tecniplast, Buguggiate, VA, Italy) for four consecutive days. The first day was used to adapt the rats to the cages, and the following 3 days were used to assess renal function. Food consumption and water intake were monitored daily. Urine samples collected during each 24-h period were used to determine urine output, creatinine, sodium and protein excretion. At the end of the experiment, terminal arterial blood samples were collected from the abdominal aorta and transferred into vacutainer tubes (BD vacutainer® SST® II Advance®, Franklin Lakes, NJ) to obtain serum. Serum was separated by centrifugation at 4000 rpm for 15 min for the measurement of biochemical parameters. Urine output was measured gravimetrically. Creatinine clearance was used to estimate GFR. Creatinine concentration was measured by the kinetic alkaline picrate (Jaffe) reaction (LabTest, Lagoa Santa, MG, Brazil). Serum and urine sodium concentrations were measured by electrolyte analyzer (ABL800 FLEX, Radiometer Copenhagen). Total urinary protein excretion was measured using a commercially available kit based on the pyrogallol red-molybdate method (Sensiprot, Labtest). All experiments were performed following the manufacturer's instructions.

### Determination of active GLP-1 serum levels

For the quantitative determination of active GLP-1, blood was collected and transferred to vacutainer collecting tubes containing the DPPIV inhibitor P32/98 (10 μM) (Abcam). Serum levels of active GLP-1 (7–36) were measured by ELISA (Glucagon-Like Peptide-1 (Active) ELISA, Merck Millipore) according to the manufacturer's instructions.

### Measurement of blood glucose

Sham and HF rats treated with vehicle or vildagliptin were fasted for 8 h. The *blood glucose* level was *determined from their tail vein* using the ACCU-CHECK® Performa meter (Roche Diagnostics GmbH, Mannheim, Germany).

### Biometric and morphometric analysis

Anesthetized rats (ketamine and xylazine 50 mg/kg and 10 mg/kg, respectively, *i.p*.) were killed by decapitation, and their hearts, kidneys and lungs were immediately removed and weighed. The heart and kidney weight to body weight ratio were used as an index of organ hypertrophy. The relative water content of lung tissue was calculated using the following equation: Lung water content (in %) = (lung wet weight − lung dry weight)/lung wet weight × 100.

The apex of the heart of each rat was separated and prepared for histological and immunohistochemical assays. The remainder of the heart was frozen at −80⋅C for molecular analysis. Five-micro meter sections of paraffin-embedded tissue were mounted onto slides and stained with hematoxylin and eosin (for determination of nuclear cell volume) or picrosirius red (for the determination of interstitial collagen density). A computerized image acquisition system (Leica Imaging Systems, Bannockburn, IL) was used for the analyses. As an estimate of myocyte hypertrophy, the average nuclear volume was determined in 70−90 cardiomyocytes cut longitudinally, acquired in 5 randomized 400 × magnification fields per each animal, and calculated according to the following equation: nuclear volume = π × D × d^2^/6 (d = shorter nuclear diameter; D = longer diameter) (Gerdes et al., 1994; dos Santos et al., 2013). Interstitial fibrosis in the remodeled LV was evaluated as the percent area occupied by collagen fibers, excluding stained ablation scars and perivascular fibers. After digitalization, the red-stained areas were quantified as the average percentage of the total area from each of five randomized 200 × magnification fields per animal. The observer was blinded to the experimental groups. Sections from the midventricular level of each heart (4 μm) stained with picrosirius red were scanned, and the circumference of the fibrotic infarct area was measured with Image J software (NIH). The size of the injured myocardial area was determined by calculating the percent of the length of the area occupied by the scar at the midventricular section. In this regard, we previously demonstrated that this single measure is a satisfactory prediction of average infarct extension (dos Santos et al., 2008).

Immunohistochemical staining was performed to measure *capillary density* and to define the localization of DPPIV in the heart. Endogenous peroxidase activity was blocked by 3 min incubation in 3% H_2_O_2_ (seven times at room temperature) and then rinsed with PBS (137 mM NaCl, 2.5 mM KCl, 10 mM Na_2_HPO_4_, and KH_2_PO_4_ 176 mM, pH 7.4). Non-specific reactions were blocked in 2% goat serum for 20 min and then incubated with the primary antibodies. The primary antibodies used were the mAb anti-DPPIV antibody or the rabbit polyclonal anti-CD31 antibody, and both of them were diluted 1:50 in the blocking buffer containing 5% BSA. Negative controls were not incubated with primary antibodies. After 18 h incubation at 4⋅C, tissues were washed 3 times for 5 min with PBS and incubated with secondary antibody. After washing in PBS, tissue sections were incubated with an HRP solution Universal LSAB 2 kit containing biotin-streptavidin complex for signal amplification of the primary antibody. Immunoreactions were detected with 3,3′-diaminobenzidine tetrahydrochloride (DAB) for 7 min. Immunostaining was visualized under a microscope and positive staining (brown color) analyzed under 400 × magnification. For capillary density evaluation, the number of capillaries CD31+ was counted from 10 randomized fields per animal at 400 × magnification. Image analysis software (Leica Imaging Systems, Bannockburn, IL, USA) was used to measure the capillary density, calculated as the number of capillaries per tissue area in the remote LV wall. The measured total tissue area was corrected for the remaining interstitial space.

### Determination of DPPIV activity and abundance

DPPIV activity was assayed in rat serum, kidney and heart homogenates using a colorimetric method that measures the release of p-nitroaniline resulting from the hydrolysis of glycylproline p-nitroanilide tosylate (Pacheco et al., 2011). Renal and heart DPPIV activity was normalized to total protein levels, and DPPIV abundance in the rat kidney and heart homogenates were analyzed by immunoblotting.

### Protein extraction from heart and renal cortex

Harvested hearts from rats were homogenized in a Polymix PX-SR 50 E homogenizer (Kinematica, AG, Switzerland) in ice-cold phosphate buffered saline (PBS) (10 mmol/L phosphate, 140 mmol/L NaCl, pH 7.4), including phosphatase inhibitors (15 mM NaF and 50 mM sodium pyrophosphate) and Halt Protease Inhibitor Cocktail (Thermo Fisher Scientific, Rockford, IL). Renal cortical homogenates were prepared as previously described (Crajoinas et al., 2014).

### Determination of protein kinase A (PKA) activity in renal cortical homogenates

Equal amounts (25 μg) of renal cortical homogenates were resolved by SDS-PAGE and analyzed by immunoblotting using an antibody specific for phosphorylated PKA substrates (Gronborg et al., 2002; Crajoinas et al., 2014).

### SDS-page and immunoblotting

Equal protein amounts of heart, renal cortical homogenate or a volume of urine containing 25 μg of creatinine were solubilized in SDS sample buffer (2% SDS, 10% glycerol, 0.1% bromophenol blue, 50 mmol/L Tris, pH 6.8), and subjected to 7.5 or 10% SDS-PAGE polyacrylamide gel. The separated proteins were transferred from the gel to a polyvinylidene difluoride membrane (PVDF) (Immobilon-P, Merck Millipore, Darmstadt, Germany) at 350 mA for 8–10 h at 4⋅C with a TE 62 Transfer Cooled Unit (GE HealthCare, Piscataway, NJ, USA), and stained with Ponceau S. PVDF membranes containing transferred proteins were subsequently blocked with 5% non-fat dry milk or 5% bovine serum albumin and 0.1% Tween 20 in PBS at a pH of 7.4 for 1 h to block non-specific binding of the antibody, followed by overnight incubation in the primary antibody. The membranes were then washed five times in blocking solution and incubated for 1 h at room temperature with an appropriated horseradish-peroxidase-conjugated immunoglobulin secondary antibody (1:2000). After washing five times in blocking solution and twice in PBS (pH 7.4), the protein bands were detected using enhanced chemiluminescence system (GE Healthcare) according to the manufacturer's protocols. The visualized bands were digitized using an ImageScanner (GE HealthCare) and quantified using the Scion Image Software package (Scion Corporation, Frederick, MD). Gels containing samples of urine were silver stained using ProteoSilver Plus Kit.

### Quantitative real time RT-PCR

Total RNA was isolated from hearts using Trizol (Thermo Fisher Scientific, Carlsbad, CA) according to the manufacturer's instructions, quantified (ND-1000 spectrophotometer—NanoDrop Technologies, Inc.), and treated with DNase-I. First-strand cDNA synthesis was performed using Super-Script III Reverse Transcriptase (Invitrogen) following the manufacturer's guidelines. The oligonucleotide primers CCAACTCCAGAGGACAACCT (forward) and TCTTCGTCCGTGTACCACAT (reverse) were used to detect DPPIV, and GATTCTGCTCCTGCTTTTCC (forward) and TCTTTTGTAGGGCCTTGGTC (reverse) were used to detect BNP. PCR products were visualized on 0.8% agarose gels with ethidium bromide. Reactions were carried using SYBR Green PCR Master Mix-PE (Thermo Fisher Scientific) on an ABI Prism® 7500 Fast Sequence Detection System (Applied Biosystem, Foster City, CA). The comparative threshold cycle method was used for data analyses. All samples were assayed in triplicate. Transcripts for three reference genes were determined: beta-actin (forward: CGTTGACATCCGTAAAGACC; reverse GCCACCAATCCACACAGA), GAPDH (forward: ATGGTGAAGGTCGGTGTG; reverse: GAACTTGCCGTGGGTAGAG) and cyclophilin A (forward: AATGCTGGACCAAACACAAA-30; reverse: CCTTCTTTCACCTTCCCAAA). The BestKeeper software (Pfaffl et al., 2004) was used to identify the best suit reference gene (Cyclophilin A) for data normalization under our experimental conditions. Relative expression was analyzed by the 2^−ΔΔ^CT method.

### Statistical analysis

All values are expressed as the means ± standard error of the mean (SEM). Comparisons between two groups were performed using unpaired *t*-tests. If more than two groups were compared, the statistical significance was determined using one-way analysis of variance (ANOVA) followed by Tukey's *post-hoc* test. The results were considered statistically significant when *p* < 0.05.

## Results

### DPPIV inhibition improves cardiac function in rats with established HF

As seen in Table 1, treatment with the DPPIV inhibitor vildagliptin reduced serum DPPIV activity by >70% in HF rats compared to HF rats treated with the vehicle.

**Table 1:** **Biometric parameters and serum DPPIV activity in sham and HF rats treated with vehicle (HF) or vildagliptin for 4 weeks**.

	**Sham**	**HF**	**HF + IDPPIV**
Initial body weight, g	252 ± 10	235 ± 8	248 ± 12
Final body weight, g	420 ± 9	441 ± 8	457 ± 9
Heart/BW, mg/g	2.55 ± 0.02	3.01 ± 0.08^***^	2.72 ± 0.05*^##^*
Lung/BW, mg/g	3.05 ± 0.07	3.56 ± 0.17^***^	3.03 ± 0.08*^###^*
Lung water content, %	78.9 ± 0.12	80.2 ± 0.2^***^	78.8 ± 0.1*^###^*
Injured myocardial area, %	−	42.1 ± 1.6	40.5 ± 1.9
Kidney/BW, mg/g	6.00 ± 0.21	6.16 ± 0.24	6.09 ± 0.19
Serum DPPIV Activity, OD	0.373 ± 0.02	0.659 ± 0.03^***^	0.186 ± 0.010^***^, *^###^*

*Values are means ± SEM. BW, body weight; OD, optical density*.

*** *p < 0.001 vs. Sham*.

## p < 0.01 and

### *p < 0.001 vs. HF*.

**Figure 1:**
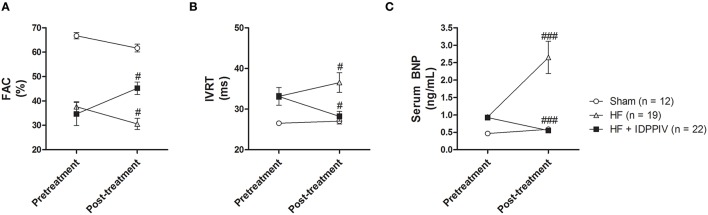
**Treatment with vildagliptin improves cardiac function in rats with established HF**. Doppler echocardiography and quantitative determination of serum BNP were performed 6 weeks after LV-radiofrequency ablation or sham surgery (pretreatment) and after 4 weeks of treatment with vehicle or vildagliptin (post-treatment). **(A)** Fractional area change (FAC). **(B)** Isovolumic relaxation time (IVRT). **(C)** Serum BNP 32 levels. Values are means ± SEM. ^#^*p* < 0.05 and ^*###*^*p* < 0.001 vs. pretreatment.

At the end of the 4-week treatment period (post-treatment), cardiac dysfunction was aggravated in HF rats who received the vehicle compared to the pretreatment period (Figure 1). Worsening of cardiac dysfunction in these animals were evidenced by a decline in FAC (37 ± 2 vs. 30 ± 2%, *p* < 0.05) (Figure 1A), which reflects a progressive reduction in the overall LV contractility, as well as an increase in IVRT (33.1 ± 0.7 vs. 36.5 ± 2.4 ms, *p* < 0.05) (Figure 1B), which reflects an aggravated diastolic dysfunction. Additionally, vehicle-treated HF rats displayed a remarkable increase in BNP serum levels from pretreatment to post-treatment (0.94 ± 0.01 vs. 2.65 ± 0.46 ng/mL *p* < 0.001). Conversely, HF rats treated with the DPPIV inhibitor vildagliptin exhibited an increase in FAC (34 ± 5 vs. 45 ± 3%, *p* < 0.05) (Figure 1A) and a reduction in IVRT (33 ± 2 vs. 27 ± 1 ms, *p* < 0.05) (Figure 1B) and in serum BNP 32 levels (0.93 ± 0.07 vs. 0.55 ± 0.02 ng/mL, *p* < 0.001) (Figure 1C) compared with the pretreatment period. As expected, cardiac function and serum BNP 32 levels were similar between pretreatment and post-treatment periods in sham rats.

### DPPIV inhibition attenuates cardiac remodeling and increases capillary density in rats with established HF

The biometric characteristics of the rats are shown in Table 1. Average body weight gain was similar among the three experimental groups. Vildagliptin administration did not influence the size of the injured myocardial area. Vehicle-treated HF rats showed higher heart weight-to-body weight ratio, indexed lung mass and the percent of water content when compared with sham-operated controls. In vildagliptin-treated rats, the heart weight-to-body weight ratio was lower than in vehicle treated HF rats but remained higher than in sham rats. The relative water content of lung tissue in vildagliptin treated HF rats was reduced to sham levels.

**Figure 2:**
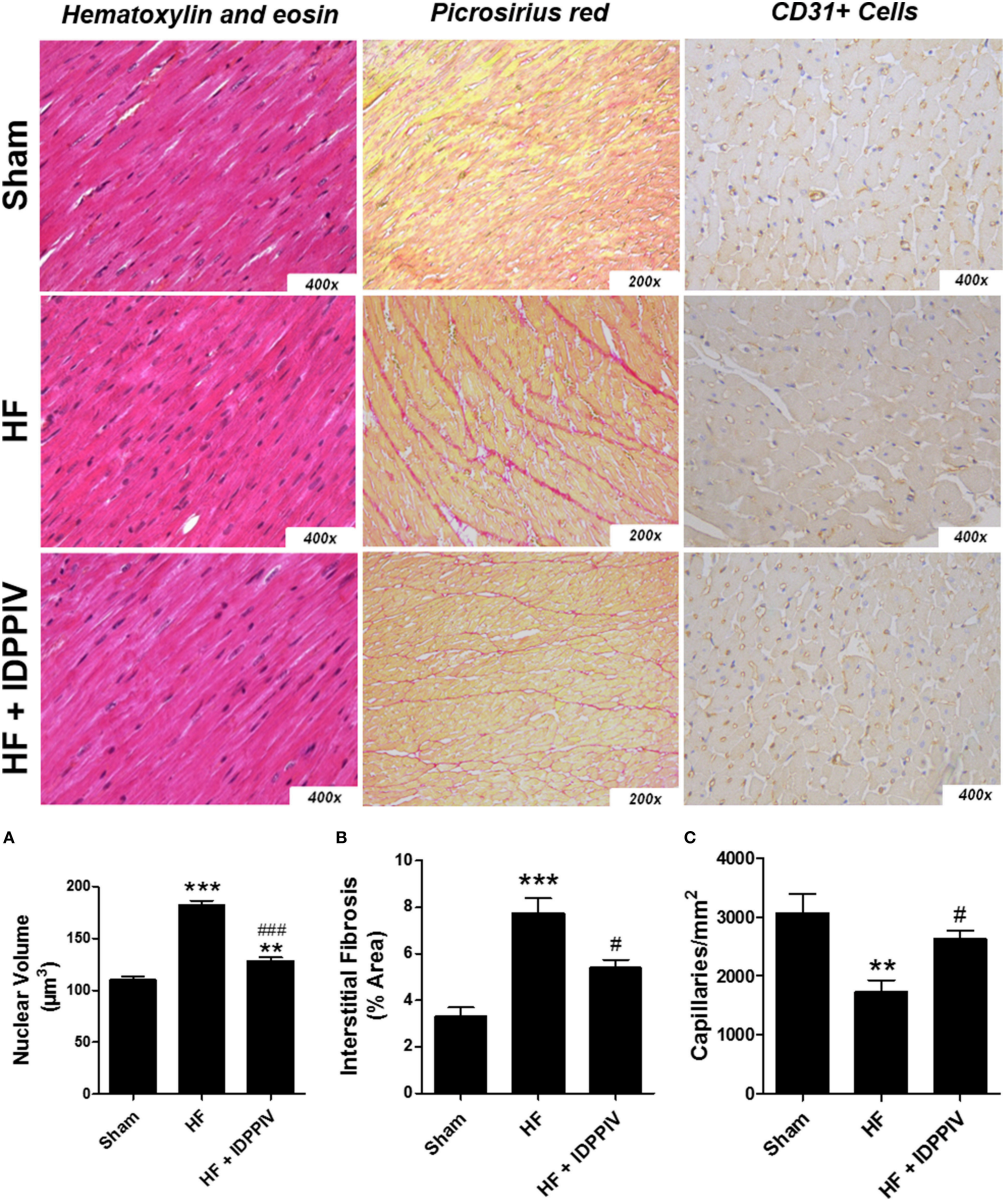
**DPPIV inhibition attenuates cardiac remodeling and capillary rarefaction in rats with established HF. (A)** Myocardial hypertrophy was assessed in HF rats treated with vehicle (HF) or vildagliptin (HF + IDPPIV) by measuring cardiomyocyte nuclear volumes in heart sections treated with stained with hematoxylin-eosin (400 × original magnification). **(B)** Cardiac interstitial fibrosis was evaluated in heart sections stained with picrosirius red (200 × original magnification). **(C)** Representative immunohistochemical staining of CD31 in sections of the LV viable wall showing individual capillaries (small dark circles) (400 × original magnification). *n* = 7–9 rats/group. Values are means ± SEM. ^**^p < 0.01 and ^***^p < 0.001 vs. Sham. ^#^*p* < 0.05 and ^*###*^*p* < 0.001 vs. HF.

The potential anti-hypertrophic effects of DPPIV inhibition in rats with established HF were further evaluated by histological analysis of hematoxylin and eosin-stained cardiac sections (Figure 2A). The results of these analyses demonstrated that the mean cardiomyocyte nuclear volume in vehicle-treated HF rats was larger than that of sham- and vildagliptin-treated rats, which significantly attenuated this increase (Figure 2A). In addition, vehicle-treated HF rats had a higher percentage of interstitial collagen in the reminiscent myocardium than sham rats, which was significantly attenuated by DPPIV inhibition (Figure 2B). Representative photomicrographs of CD31 stained sections in the LV viable wall showing individual capillaries are presented in the Figure 2C. HF rats treated with vildagliptin showed a slight but significant increase in the amount of CD31+ capillaries compared to the vehicle-treated group in the remote area of injured hearts indicating a reduced capillary rarefaction. Moreover, the non-treated HF rats had a significant decrease in the number of capillaries per mm^2^ compared to sham group (Figure 2C).

### DPPIV inhibition suppresses cardiac DPPIV activity and expression in rats with established HF

**Figure 3:**
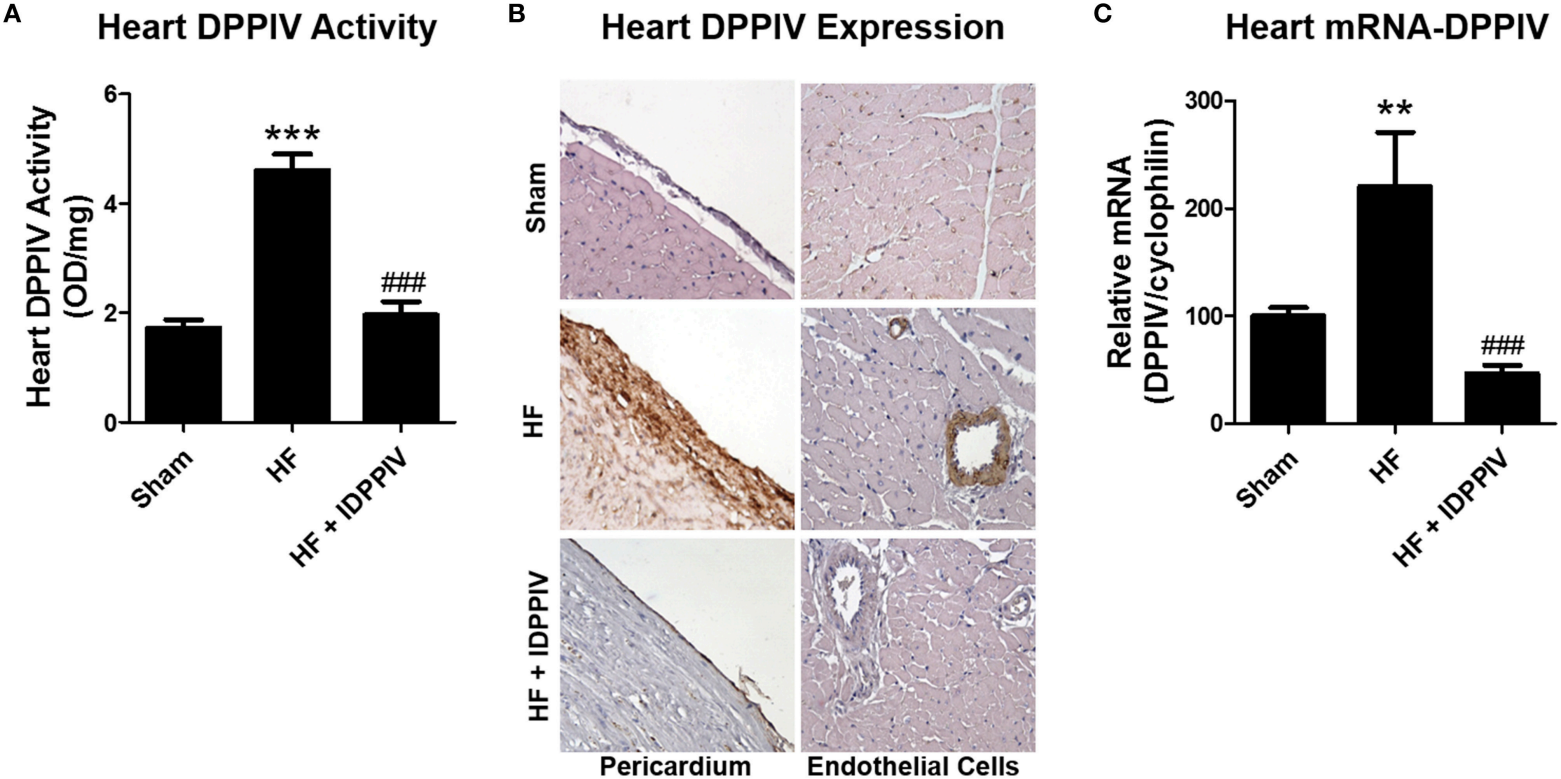
**Heart DPPIV activity and expression in sham and HF rats treated with the DPPIV inhibitor vildagliptin or with the vehicle. (A)** Heart DPPIV activity was measured in sham and HF rats treated with vehicle (HF) or vildagliptin (HF + IDPPIV) by colorimetry. **(B)** Representative immunohistochemical staining of DPPIV in the heart (400 × original magnification). **(C)** Graphical representation of the relative gene expression of DPPIV in the heart of sham and HF rats treated with vildagliptin or with the vehicle. The levels of mRNA of DPPIV were measured by real-time PCR and cyclophilin used as an internal control. Values are means ± SEM. ^**^p < 0.01 and ^***^p < 0.001 vs. Sham. ^*###*^*p* < 0.001 vs. HF.

Vehicle-treated HF rats exhibited higher levels of cardiac DPPIV activity compared to sham and vildagliptin, which markedly decreased DPPIV activity (Figure 3A). Increased heart DPPIV expression in HF rats was observed both in cardiac endothelial cells as well as in the pericardium membrane (Figure 3B). Notwithstanding, vildagliptin reduced DPPIV expression at both sites. Higher DPPIV activity and protein expression was accompanied by higher levels of DPPIV-mRNA expression in the heart of HF rats compared to sham rats, which was also attenuated by vildagliptin treatment (Figure 3C).

### DPPIV inhibition diminishes DPPIV activity but not expression in the kidney of rats with established HF

**Figure 4:**
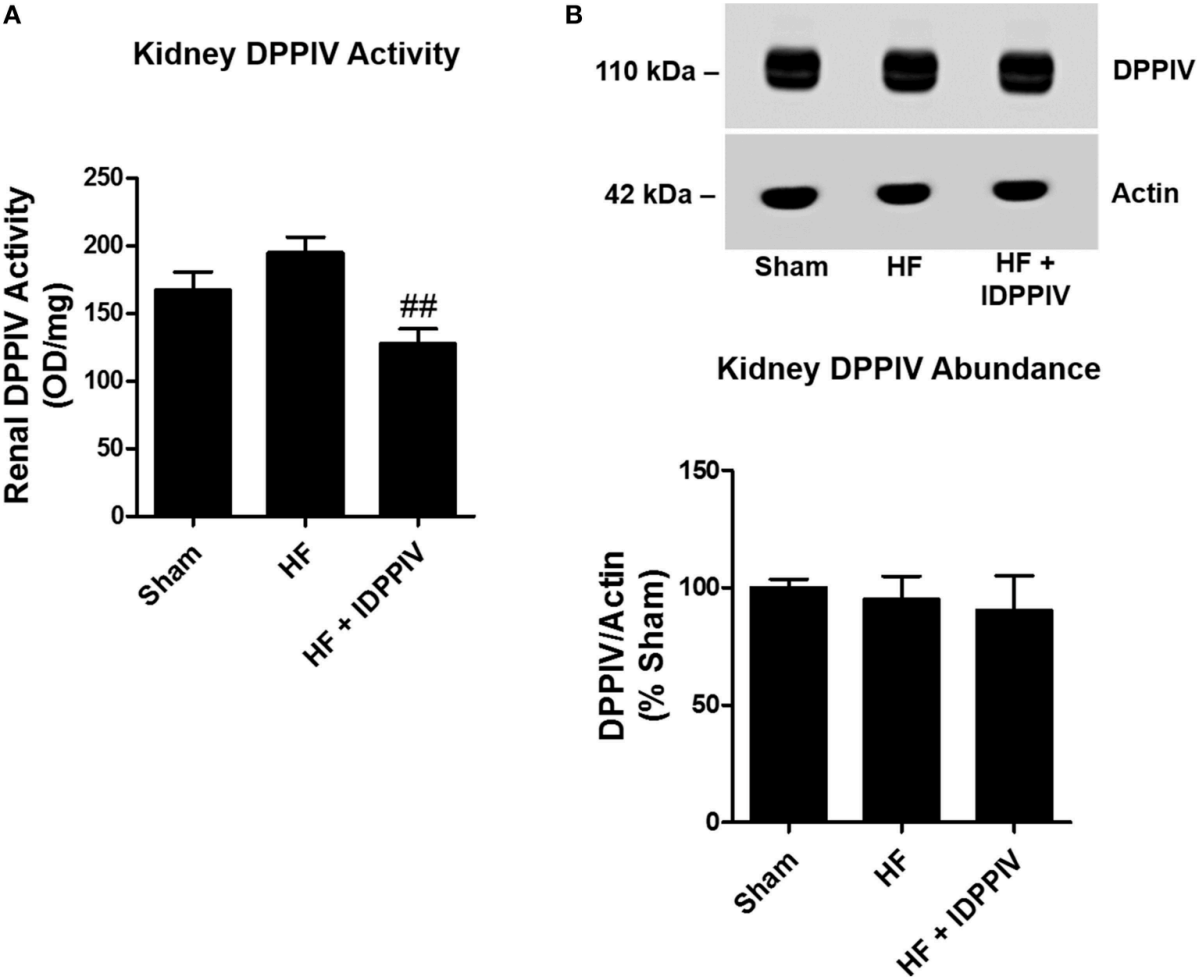
**Renal DPPIV activity and expression in sham and HF rats treated with the DPPIV inhibitor vildagliptin or with the vehicle. (A)** Renal DPPIV activity was measured in sham and HF rats treated with vehicle (HF) or vildagliptin (HF + IDPPIV) by colorimetry. **(B)** Renal cortical DPPIV abundance was evaluated by immunoblotting. Equal amounts of protein (10 μg for DPPIV and 5 μg for actin) were subjected to SDS-PAGE, transferred to a PVDF membrane and incubated with the following primary antibodies: anti-DPPIV (1:1000) and anti-actin (1:50,000). Membranes were stained with Ponceau S prior to antibody incubation, and the Actin was used as an internal control. Values are means ± SEM. ^*##*^*p* < 0.01 vs. HF.

The activity and protein expression of DPPIV in the renal cortex of sham and HF rats treated with vildagliptin or with the vehicle are illustrated at the Figure 4. Renal cortical DPPIV activity was lower in HF rats treated with vildagliptin compared to both sham and vehicle-treated HF rats (Figure 4A). As shown in Figure 4B, the expression of DPPIV in the renal cortex was similar among the three groups of rats.

### DPPIV inhibition increases circulating GLP-1 active and restores PKA signaling in the kidneys of rats with established HF

**Figure 5:**
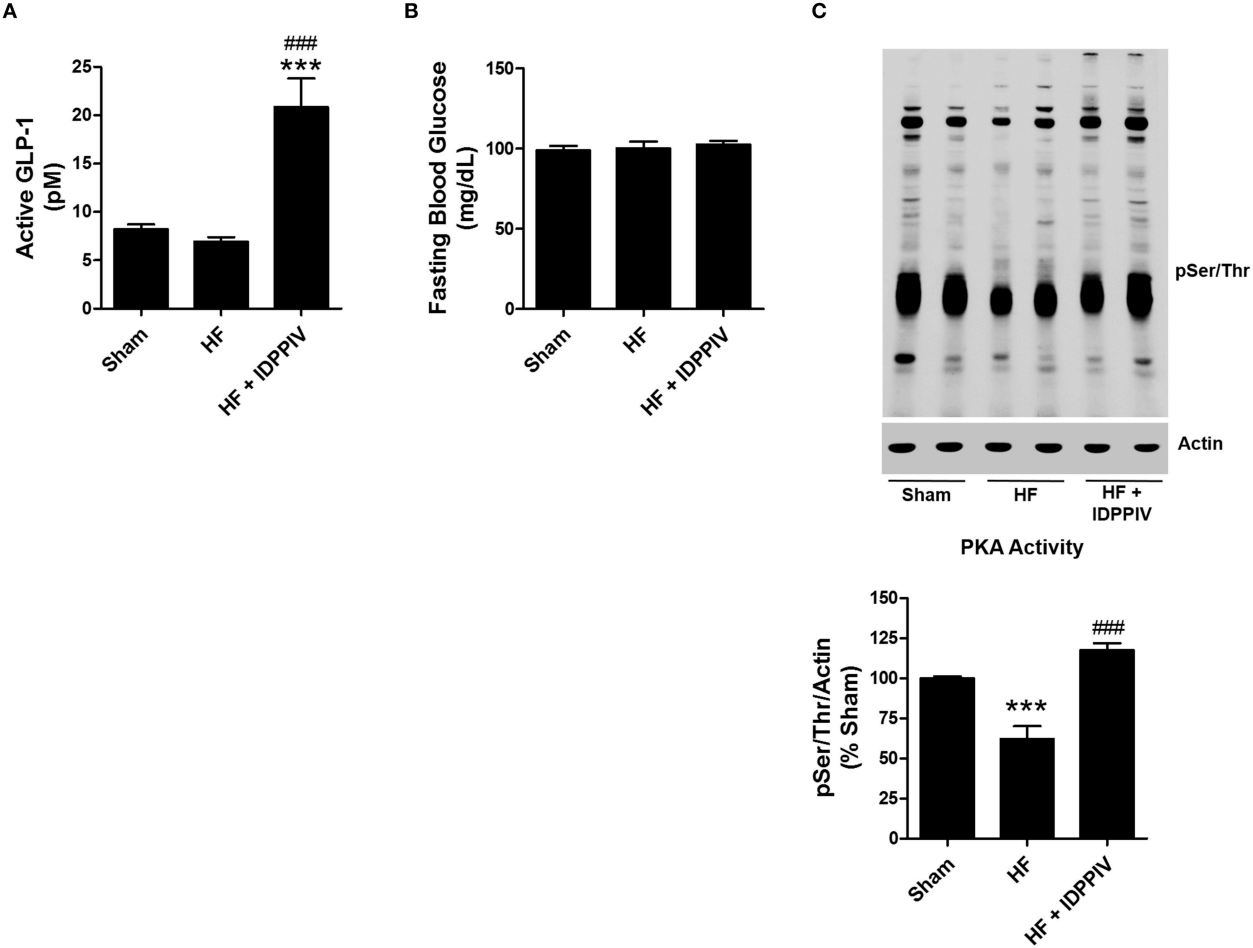
**DPPIV inhibition increases active GLP-1 serum concentration as well as renal cortical GLP-1R expression and PKA activation in HF rats. (A)** Serum levels of active GLP-1 were determined by ELISA in sham and HF rats treated with vildagliptin (HF + IDPPIV) or vehicle (HF). **(B)** Fasting glucose serum levels in sham, HF rats and HF rats treated with vildagliptin. *n* = 11–17 rats/group **(A,B)**. **(C)** Levels of phosphorylation of PKA substrates in the renal cortex of Sham, HF and HF rats treated with vildagliptin (HF + IDPPIV) were evaluated by immunoblotting using an antibody that recognizes proteins containing a phospho-Ser/Thr. Top: Equal amounts of protein (25 μg) from each rat were subjected to SDS-PAGE, transferred to a PVDF membrane and incubated with the antibody anti-pSer/Thr. Actin was used as an internal control. Bottom: The sum total of all phospho-PKA proteins per lane was estimated by densitometry and normalized by actin. The combined data from 6 experiments are represented as columns in a bar graph. Values are means ± SEM. ^***^p < 0.001 vs. Sham. ^*###*^*p* < 0.001 vs. HF.

The levels of active GLP-1 in HF rats treated with vildagliptin (20.9 ± 2.9 pM) were approximately three times higher than those of vehicle-treated HF rats (6.9 ± 0.4 pM *p* < 0.001) and sham rats (8.2 ± 0.5 pM *P* < 0.001) (Figure 5A). However, despite the differences in GLP-1 levels between vehicle-treated-HF and HF rats treated with vildaglitpin, the *fasting blood glucose* levels were similar among the groups (Figure 5B).

The effect of DPPIV inhibition on renal cortical PKA activity, a downstream effector for the GLP-1R (Farah et al., 2016), was estimated by immunoblotting. As shown in Figure 5C, vehicle-treated HF rats exhibited lower renal cortical PKA activity than sham rats. Vildagliptin treatment increased the levels of phosphorylated PKA substrates in the renal cortex of HF rats compared to vehicle-treated HF rats.

### DPPIV inhibition improves renal sodium and water handling in rats with established HF

**Figure 6:**
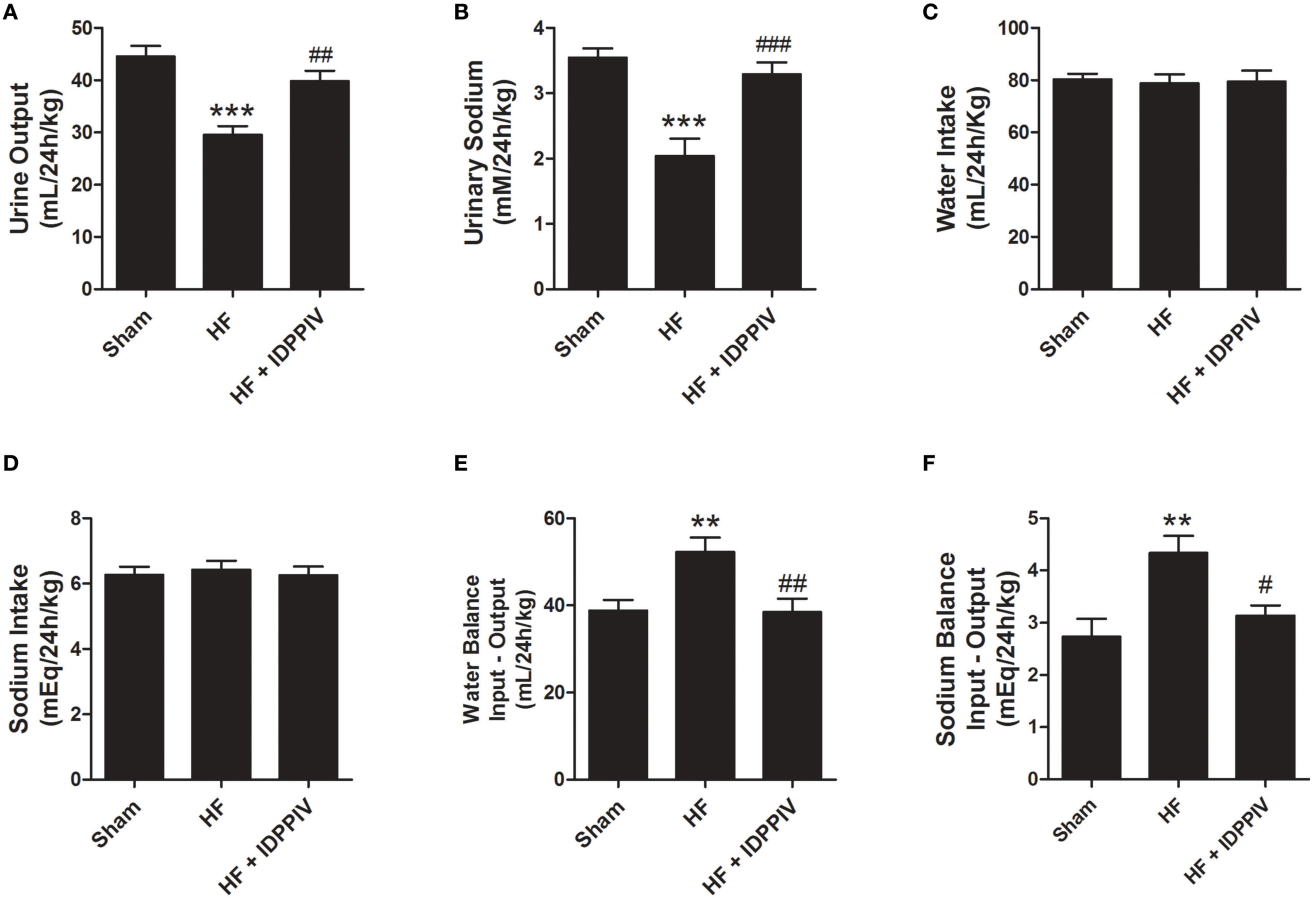
**DPPIV inhibition by vildagliptin improves renal handling of sodium and water of HF rats**. Sham and HF rats treated with vildagliptin (HF + IDPPIV) or vehicle (HF) were individually placed into metabolic cages for 24-h urine collection for three consecutive days to evaluate **(A)** urinary flow and **(B)** urinary Na^+^ excretion. **(C)** Water and food consumption were measured daily. **(D)** Sodium intake was calculated based on the sodium content of the rodent chow and on the daily consumption of chow by each rat. **(E)** Water and **(F)** sodium balance. *n* = 11–17 rats/group. Values are means ± SEM. ^**^p < 0.01 and ^***^p < 0.001 vs. Sham. ^#^*p* < 0.05, ^*##*^*p* < 0.01, ^*###*^*p* < 0.001 vs. HF.

**Figure 7:**
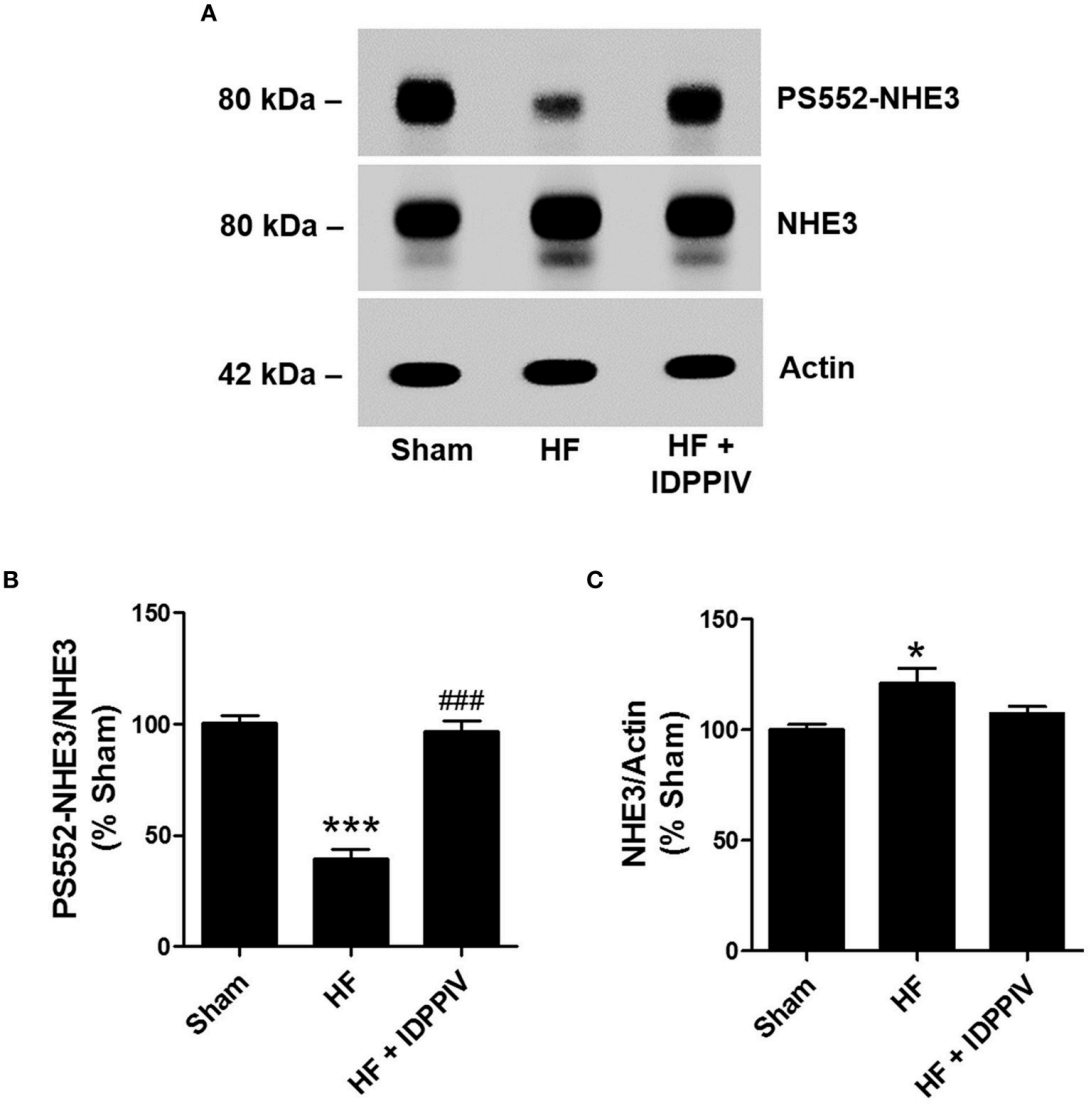
**Treatment with vildagliptin increases NHE3 phosphorylation at the PKA consensus site serine 552 in the renal cortex of HF rats. (A)** Representative immunoblotting of renal cortical proteins probed with a phosphospecific NHE3 mAb that recognizes NHE3 only when it is phosphorylated at serine 552 (PS552-NHE3), total NHE3 mAb or anti-actin. **(B)** Graphical representation of the relative PS552-NHE3 and **(C)** total NHE3 levels. The relative abundance of PS552-NHE3 was quantitated by densitometry and expressed as a ratio of PS552-NHE3/total. The combined data from 4 experiments are represented. Values are means ± SEM. ^*^p < 0.05 and ^***^p < 0.001 vs. Sham. ^*###*^*p* < 0.001 vs. HF.

The effects of DPPIV inhibition on sodium and water handling are shown in Figure 7. HF rats exhibited lower urinary flow (Figure 6A) and sodium excretion (Figure 6B) compared to sham rats, whereas treatment with vildagliptin significantly restored urine output and sodium excretion in rats with established HF (Figures 6A,B). The mean values of water and sodium intake were not significantly different among the three experimental groups of rats (Figures 6C,D). Accordingly, HF rats treated with the vehicle exhibited higher positive water (Figure 6E) and sodium balance (Figure 6F) compared to sham rats. Daily water and sodium balance in HF rats treated with vildagliptin were similar to sham rats and lower than non-treated HF rats.

### DPPIV inhibition increases NHE3 phosphorylation at serine 552 in the renal cortex of HF rats

Lower levels of phosphorylated NHE3 at the PKA consensus site serine 552 in the proximal tubule has been associated with higher NHE3 transport activity (Crajoinas et al., 2010, 2014; Girardi and Di Sole, 2012; Pontes et al., 2015; Farah et al., 2016). As show in Figures 7A,B, the levels of NHE3 phosphorylation at serine 552 (PS552-NHE3) were much lower in HF than in sham-operated rats (39 ± 4 vs. 100 ± 3%, *P* < 0.01) (Figures 7A,B). In rats treated with vildagliptin, PS552-NHE3 was higher than in vehicle treated HF rats and similar to sham rats. Total NHE3 protein expression was slightly higher in the renal cortex of vehicle-treated HF rats than in sham-operated rats and vildagliptin-treated HF rats (Figures 7A,C).

### DPPIV inhibition improves GFR and exerts anti-proteinuric effects in rats with established HF

**Figure 8:**
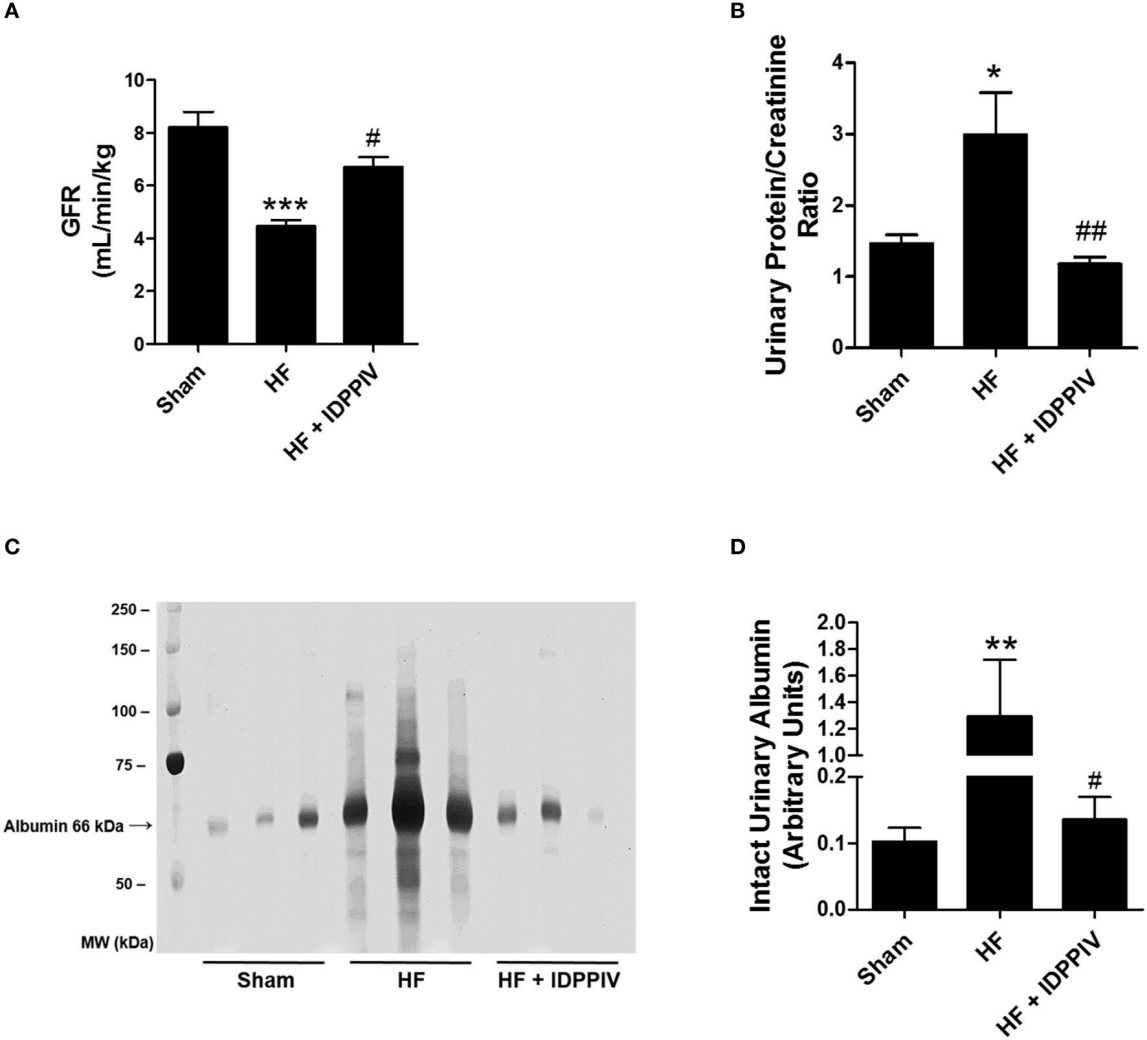
**DPPIV inhibition improves GFR and reduces proteinuria in HF rats. (A)** The glomerular filtration rate (GFR) was estimated by the creatinine clearance. **(B)** Urine protein to creatinine ratio. *n* = 11–17 rats/group. **(C)** Profile of urinary proteins excreted by sham and HF rats treated with vehicle (HF) or vildagliptin (HF + IDPPV). The 24-h urine samples (volume equivalent to 25 μg of creatinine) were subjected to 10% SDS-PAGE. Following electrophoresis, the gels were silver stained using the ProteoSilver Plus Kit (Sigma-Aldrich). **(D)** Graphic representation of the amount of intact albumin semiquantitatively evaluated by densitometry. *n* = 6 rats/group. Values are means ± SEM. ^*^p < 0.05, ^**^p < 0.01 and ^***^p < 0.001 vs. Sham. ^#^*p* < 0.05, ^*##*^*p* < 0.01 vs. HF.

As depicted in Figure 8, vehicle-treated HF rats exhibited a much lower GFR (Figure 8A) and a much higher excretion of protein in the urine than sham rats (Figures 8B,C). Vildagliptin treatment restored both GFR and proteinuria to sham levels. These changes on GFR were not accompanied by changes on the kidney/body weight ratios (Table 1). The profile of urinary proteins excreted by HF and sham rats was evaluated by SDS-PAGE, and the amount of intact albumin was semiquantitatively determined by densitometry. As seen in Figures 8C,D, the urinary excretion of intact albumin was remarkably higher in vehicle-treated HF rats compared to both sham and HF rats treated with vildagliptin. Moreover, the levels of albumin excretion were not significantly different between vildagliptin-treated HF rats and sham rats. Analysis of the pattern of proteinuria excreted by vehicle-treated HF rats (Figure 8C) suggests a mixed origin of proteinuria because both glomerular and tubular protein fractions, i.e., high and low molecular weight proteins, were present in the urine.

### DPPIV inhibition increases renal cortical expression of megalin, nephrin and podocin expression in rats with established HF

**Figure 9:**
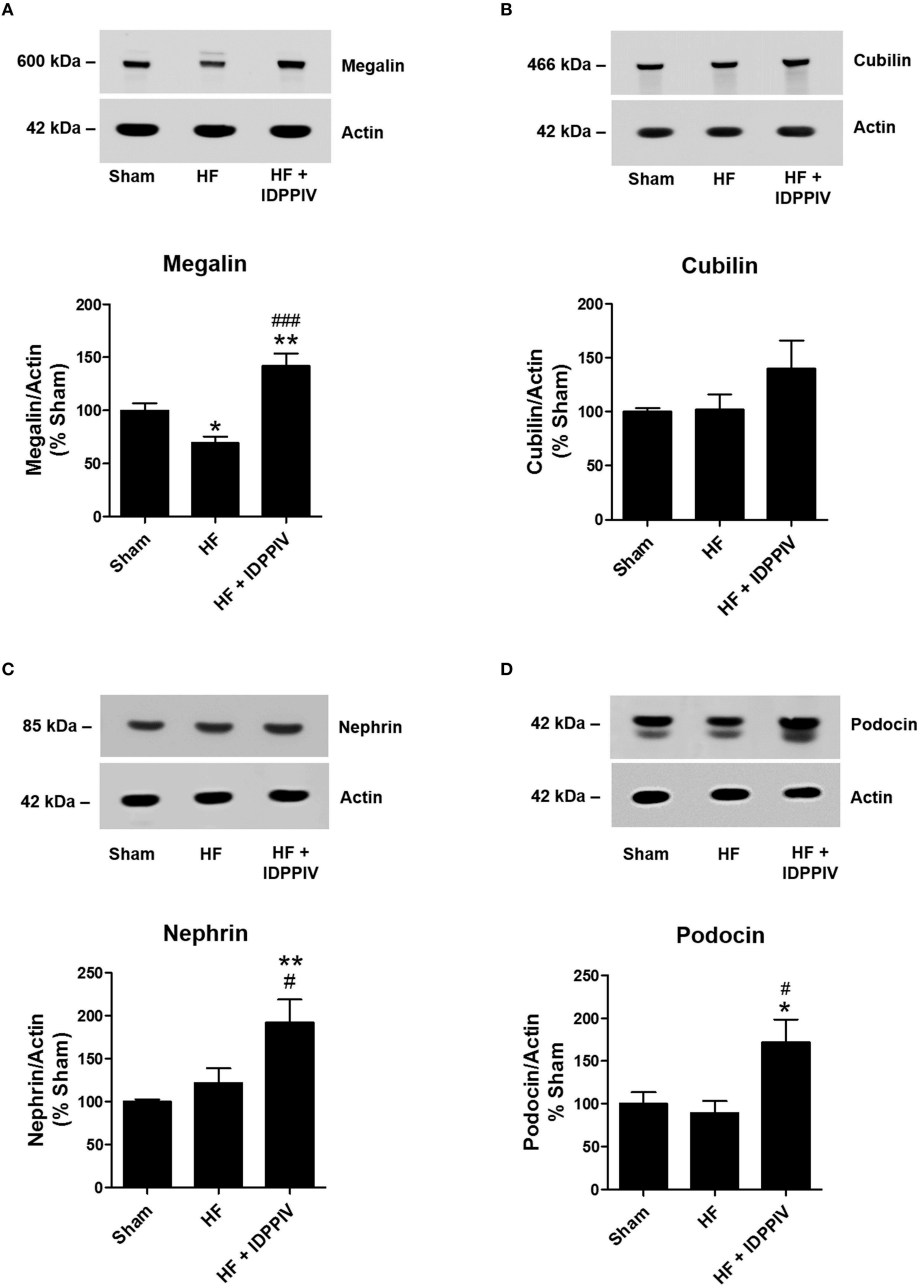
**The antiproteinuric effects of DPPIV inhibition are associated with upregulation of megalin, nephrin, and podocin expressions in the kidneys of HF rats**. Equal amounts of renal cortical proteins isolated from sham and HF rats treated with vildagliptin (HF + IDPPIV) or vehicle (HF) (10 μg for megalin and cubilin, 30 μg for nephrin and podocin and 5 μg for actin) were subjected to SDS-PAGE, transferred to a PVDF membrane and incubated with primary antibodies against **(A)** megalin (1:50,000), **(B)** cubilin (1:1000), **(C)** nephrin (1:11,000), or **(D)** podocin (1:11,000). Actin was used as an internal control. Values are means ± SEM. ^*^p < 0.05 and ^**^p < 0.01 vs. Sham. ^#^*p* < 0.05, ^*###*^*p* < 0.001 vs. HF.

Given the anti-proteinuric effect of vildagliptin treatment, we next tested the hypothesis that DPPIV inhibition could regulate the expression of components of the apical endocytic machinery in the renal proximal tubule (megalin and cubilin) (Willnow et al., 1996; Leheste et al., 1999; Birn and Christensen, 2006) and/or components of the glomerular filtration barrier, including nephrin and podocin (Kestila et al., 1998; Tryggvason, 1999; Boute et al., 2000; Luimula et al., 2000; Agrawal et al., 2013). As shown in Figure 9A, the protein expression of the endocytic receptor megalin was significantly reduced in the cortex of HF rats compared to sham rats (69 ± 6 vs. 100 ± 3%, *p* < 0.05). Interestingly, vildagliptin treatment upregulated megalin to levels higher than sham rats (142 ± 11 vs. 100 ± 3%, *p* < 0.01). Conversely, the abundance of cubilin in the renal cortex was similar among the three groups of rats (Figure 9B). Although the podocyte main slit diaphragm proteins (Kestila et al., 1998; Tryggvason, 1999; Boute et al., 2000; Luimula et al., 2000; Agrawal et al., 2013), nephrin and podocin, were similar between sham and vehicle-treated HF rats, they were upregulated by DPPIV inhibition in the renal cortex of rats with established HF (Figures 9C,D).

## Discussion

The heart and the kidney are very closely related. Cardiac impairment can result in renal dysfunction, and worsening of renal function is a strong predictor of long-term adverse outcomes in patients with HF. In the present study, we demonstrated that chronic treatment with the DPPIV inhibitor vildagliptin exerts renoprotective and cardioprotective effects in rats with established HF, reversing cardiac remodeling and improving both LV systolic and diastolic function. Long-term renoprotection conferred by DPPIV inhibition involves improved renal handling of sodium and water which may have ultimately led to relief of volume expansion and pulmonary congestion in HF. Additionally, DPPIV inhibition significantly reduces proteinuria in HF rats. The anti-proteinuric effects of DPPIV inhibition were associated with upregulation of the apical proximal tubule endocytic receptor megalin as well as of the podocyte main slit diaphragm proteins nephrin and podocin.

We have recently reported (dos Santos et al., 2013) that humans and rats with HF exhibit higher plasma levels of DPPIV activity and abundance compared to their healthy counterparts. Additionally, previous studies from our laboratory (dos Santos et al., 2013) and others (Sauvé et al., 2010; Gomez et al., 2012; Shigeta et al., 2012; Aoyama et al., 2016) have demonstrated that genetic deletion or pharmacologic inhibition of DPPIV prevents the onset of HF after myocardial infarct/injury in rodents and large animal models. However, to the best of our knowledge, this is the first report that reveals that DPPIV inhibition can exert not only preventive but also therapeutic effects in HF by restoring myocardial structure and function. Our results that vildagliptin ameliorated cardiorenal function in rats with established HF contrast with those from Yin et al. (2011). These authors did not observe any beneficial effect of vildagliptin at a daily dose of 15 mg/kg/day once daily on either preventing or reversing cardiac remodeling and dysfunction in post-myocardial infarcted rats. One plausible explanation of this discrepancy between our findings and theirs is the higher dose (120 vs. 15 mg/kg/day) and higher frequency of administration of vildagliptin we employed (twice daily vs. once daily dosing). Indeed, we have noticed that although chronic treatment with 20 mg/kg/day vildagliptin inhibits plasma DPPIV activity, it fails to inhibit the activity of the peptidase in the heart and in the kidneys of HF rats. Moreover, at this low dose, vildagliptin is unable to ameliorate cardiac and renal function and to reduce pulmonary congestion in rats with established HF (unpublished observations). In concert, these observations suggest that local inhibition of DPPIV activity in the heart and possibly in the kidney may have a pivotal role in mediating the therapeutic effects of DPPIV inhibitors in rats with HF.

An intriguing finding of the present work is that administration of vildagliptin, a competitive inhibitor of DPPIV catalytic activity, not only inhibits the activity of DPPIV but also reduces the protein and mRNA-expression of the peptidase in the heart of HF rats. Similarly, Kanasaki et al. (2014) have recently reported that administration of the DPPIV inhibitor linagliptin reduces DPPIV activity and expression in the kidney and endothelial cells of streptozotocin-induced diabetic mice. Reduced DPPIV expression in these diabetic mice was associated with an upregulation of components of the microRNA (miRNA) 29 family. Interestingly, the miRNA 29 family has been shown to be downregulated after myocardial infarction (van Rooij et al., 2008; Melo et al., 2014), and this downregulation contributes to cardiac fibrosis (van Rooij et al., 2008). However, it remains to be established whether this post-transcriptional mechanism is also involved in the upregulation of DPPIV activity/expression in the heart of experimental models of HF.

The cardioprotective effects of DPPIV inhibition are often attributed to increased bioavailability of GLP-1, BNP, and SDF-1α that ameliorate cardiac performance and contractility, reduce hypertrophy, fibrosis and apoptosis, and improve stem cell mobilization and angiogenesis to the myocardium (Zaruba et al., 2009; Shigeta et al., 2012; dos Santos et al., 2013; Hocher et al., 2013). The present data suggest a role for the renoprotective actions of vildagliptin in the outcomes of rats with established HF. Indeed, it is undeniable that neurohumoral activation in response to the low output in decompensated HF and the consequent water and salt retention by the kidneys are important factors that increase circulatory filling pressure and restore cardiac ejection (Cadnapaphornchai et al., 2001; Chatterjee, 2005; Brum et al., 2006). However, chronic and excessive volume expansion with increased preload can lead to cardiac remodeling and dilation, which has deleterious effects on cardiac function. In fact, changes on renal function are not only a marker of HF but may also be a pathogenic factor in causing the progression of cardiac deterioration (Boerrigter et al., 2013). One may therefore speculate that improvement of cardiac remodeling and function in vildagliptin-treated HF rats may result, at least in part, from restored renal function.

Derangements in several hormonal systems contribute to sodium and water retention in HF. The results from our previous (dos Santos et al., 2013) and present work suggest that restoration of the GLP-1/GLP-1R signaling activation in the renal cortex may be implicated in the vildagliptin-mediated amelioration of renal function in HF rats. The acute diuretic and natriuretic actions of GLP-1 have been consistently demonstrated by a variety of studies in rodents (Moreno et al., 2002; Crajoinas et al., 2011; Rieg et al., 2012; Thomson et al., 2013; Farah et al., 2016) and humans (Gutzwiller et al., 2004, 2006; Skov et al., 2013). The effects of GLP-1 on sodium and water handling in both rodents and humans is mediated, at least in part, by inhibition of NHE3-mediated renal proximal tubule sodium reabsorption via activation of the cAMP/PKA signaling pathway (Crajoinas et al., 2011; Rieg et al., 2012; Thomson et al., 2013; Farah et al., 2016). Accordingly, in the present work, we found that vildagliptin administration in rats with established HF elevated the concentration of circulating active GLP-1, and most likely, the concentration of this incretin hormone in the renal tubular fluid restores PKA activation to the levels of sham rats. These events were associated with higher levels of renal cortical NHE3 phosphorylation at the PKA consensus site serine 552, a surrogate for reduced NHE3 transport activity in the proximal tubule (Crajoinas et al., 2010; Girardi and Di Sole, 2012). It is noteworthy that GLP-1 also exerts diuretic and natriuretic effects in rodents through increments in both renal blood flow and GFR (Jensen et al., 2015). Indeed, recent studies have shown that GLP-1R is expressed in the afferent arterioles and that stimulation of GLP-1R by specific agonists increases renal blood flow (Thomson et al., 2013; Jensen et al., 2015). Therefore, our finding that HF rats treated with vildagliptin display higher GFR than vehicle-treated HF rats may also be due, at least in part, to the activation of GLP-1/GLP-1R in the renal vasculature.

DPPIV modulates multiple substrates other than GLP-1 that exert natriuretic and/or renoprotective effects, such as BNP, SDF-1α, substance P, among others (Makino et al., 2015). Thus, it is important to emphasize that the beneficial effects of DPPIV inhibition on sodium and water balance in HF shown herein might, in part, occur via GLP-1 independent mechanisms. In this context, Rieg and colleagues found that administration of the DPPIV inhibitor alogliptin was capable of inducing diuresis and natriuresis in GLP-1R knockout mice (Rieg et al., 2012). Moreover, we have previously found that inhibition of the catalytic activity of DPPIV inhibits NHE3 activity in the opossum kidney clone P (OKP) proximal tubule cell line, and it is well known that the proximal tubule does not synthesize GLP-1 (Girardi et al., 2008).

In addition to the effects on sodium and water homeostasis, pharmacological administration of DPPIV inhibitors confers renoprotection by reducing proteinuria and ameliorating renal damage in experimental models of diabetic nephropathy (Liu et al., 2012; Kanasaki et al., 2014; Eun Lee et al., 2016). The anti-proteinuric effects of DPPIV inhibition have also been observed in type 2 diabetes patients (Hattori, 2011; Groop et al., 2013; Kawasaki et al., 2015; Nakamura et al., 2016). In the present study, we found that HF rats display tubular and glomerular proteinuria and that tubular proteinuria in these animals was associated with reduced expression of the endocytic receptor megalin. On the other hand, the origin of glomerular proteinuria in these rats remains obscure. It is worth mentioning that glomerular proteinuria has not often been associated with lower expression of nephrin but instead with changes in the levels of tyrosine phosphorylation of its cytoplasmic tail (Carney, 2016; New et al., 2016). Surprisingly, treatment with vildagliptin upregulated megalin as well as the podocyte slit diaphragm proteins nephrin and podocin in the kidneys of HF rats to levels higher than sham. The molecular mechanisms by which vildagliptin increases the abundance of megalin, nephrin and podocin in the renal cortex of HF rats is yet to be established.

In summary, our findings demonstrated that the DPPIV inhibitor vildagliptin exerts renoprotective effects and ameliorates cardiorenal function in rats with established HF. Moreover, our data suggest that DPPIV may constitute one of the pathophysiological connections between the failing heart and kidneys. Given the lack of pharmacological agents that directly improve renal function in patients with HF, long-term studies with DPPIV inhibitors are warranted to ascertain whether the renoprotective effects of DPPIV inhibition ultimately translate into improved clinical outcomes.

## Author contributions

DA, performed experiments, analyzed data, interpreted the results of the experiments, prepared figures, and drafted the manuscript. FM, performed experiments, analyzed data, and interpreted the results of the experiments. RD, performed experiments, analyzed data, interpreted the results of the experiments, and edited and revised the manuscript. LS, performed experiments and analyzed data. EA, performed experiments. LD, interpreted the results of the experiments and drafted, edited and revised manuscript. PT, developed the experimental model of HF and edited and revised manuscript. AG, conceived of and designed the research, prepared figures, and drafted, edited and revised manuscript. All, approved final version of the manuscript.

### Conflict of interest statement

The authors declare that the research was conducted in the absence of any commercial or financial relationships that could be construed as a potential conflict of interest.
